# Enhancing yield prediction from plot-level satellite imagery through genotype and environment feature disentanglement

**DOI:** 10.3389/fpls.2025.1617831

**Published:** 2025-09-30

**Authors:** Anirudha A. Powadi, Talukder Z. Jubery, Michael Tross, Nikee Shrestha, Lisa Coffey, James C. Schnable, Patrick S. Schnable, Baskar Ganapathysubramanian

**Affiliations:** ^1^ Department of Electrical and Computer Engineering, Iowa State University, Ames, IA, United States; ^2^ Translational AI Research and Education Center, Iowa State University, Ames, IA, United States; ^3^ Department of Agronomy, University of Nebraska, Lincoln, NE, United States; ^4^ Department of Agronomy, Iowa State University, Ames, IA, United States; ^5^ Plant Sciences Institute, Iowa State University, Ames, IA, United States; ^6^ Department of Mechanical Engineering, Iowa State University, Ames, IA, United States

**Keywords:** representation learning, genotype × environment interactions, crop yield prediction, satellite data, latent feature extraction

## Abstract

Accurately predicting yield during the growing season enables improved crop management and better resource allocation for both breeders and growers. Existing yield prediction models for an entire field or individual plots are based on satellite-derived vegetation indices (VIs) and widely used machine learning-based feature extraction models, including principal component analysis (PCA) and autoencoders (AE). Here, we significantly enhance pre-harvest yield prediction at plot-scale using Compositional Autoencoders (CAE) — a deep-learning-based feature extraction approach designed to disentangle genotype (G) and environment (E) features — on high-resolution, plot-level satellite imagery. Our approach uses a dataset of approximately 4,000 satellite images collected from replicated plots of 84 hybrid maize varieties grown at five distinct locations across the U.S. Corn Belt. By deploying the CAE model, we improve the separation of genotype and environment effects, enabling more accurate incorporation of genotype-by-environment (GxE) interactions for downstream prediction tasks. Results show that the CAE-based features improve early-stage yield predictions by up to 10% compared to traditional autoencoder-based features and outperform vegetation indices (VIs) by 9% across various growth stages. The CAE model also excels in separating environmental factors, achieving a high silhouette score of 0.919, indicating effective clustering of environmental features. Moreover, the CAE consistently outperforms standard models in unseen environments and unseen genotypes yield predictions, demonstrating strong generalizability. This study demonstrates the value of disentangling G and E effects for providing more accurate and early yield predictions that support informed decision-making in precision agriculture and plant breeding.

## 1 Introduction

Maize (*Zea mays L.*) is one of the world’s most important cereal crops, with nearly one billion tons of maize produced on about 200 million planted hectares annually (Erenstein et al., 2022). The productivity of maize is highly variable, largely due to differences in environmental conditions (Jägermeyr et al., 2021; Yang et al., 2024), crop management practices (Lobell et al., 2014; Ort and Long, 2014; Lobell et al., 2020), and genetic factors (Gamble, 1962; Troyer, 1990; Butrón et al., 2012; Nolan and Santos, 2012). These three components interact in complex ways, complicating the task of accurately predicting yields for specific hybrids under particular conditions and crop management strategies.

Yield forecasting is crucial for multiple stakeholders in agriculture. Farmers benefit from reliable early predictions, as these help optimize management practices, maximizing the use of limited resources like water and fertilizers. Meanwhile, breeders pursue stable genotypes that perform well in diverse environments, which requires extensive testing of thousands of genotypes in dozens of locations to identify those most suitable for target regions (Cooper et al., 2014; Li et al., 2024; Tarekegne et al., 2024). Thus, improving yield prediction systems directly supports agricultural resilience and the long-term stability of food systems.

Remote sensing technologies have been extensively used to tackle the challenges of yield prediction. Historically, low-resolution field- or county-level satellite imagery has been extensively used for predictive plant phenotyping, including yield estimation for dry beans, rice, maize, wheat, soybean (Hamar et al., 1996; Yang et al., 2009; Wang et al., 2010; Noureldin et al., 2013; Johnson et al., 2016b; Jin et al., 2017; Schwalbert et al., 2018; Kamir et al., 2020; Schwalbert R. et al., 2020; Schwalbert RA. et al., 2020; Shendryk et al., 2021; Roznik et al., 2022; Joshi et al., 2023). This imagery allows researchers to gain a broader perspective on crop performance across large regions. UAVs (unmanned aerial vehicles) have emerged as a complementary tool, offering plot-level high-resolution data (López-Granados et al., 2016; Kanning et al., 2018; Han et al., 2019; Du et al., 2022), yet satellite imagery retains certain advantages, particularly for large-scale studies. It allows data collection over extensive areas with considerably less logistical effort, while also delivering preprocessed data and reducing user workload. This makes it the preferred choice for multi-location analyses. Despite this benefit, the utility of satellite data for plot-level prediction is often hindered by lower spatial resolution, which requires specialized acquisition strategies to obtain more detailed imagery (Phang et al., 2023). High-resolution satellite imagery is becoming available, although applications still face obstacles such as restricted satellite availability, limited onboard storage capacity, and cloud cover (over the target area), making high-resolution data acquisition challenging. Despite the challenges, the advantages of collecting high-resolution satellite data instead of UAV data for breeding applications may outweigh the limitations. Evaluating the large numbers of varieties (called hybrids) across many different environments becomes easier with satellite data. The most important and most common metric of evaluating hybrids is yield. The ability to forecast yield earlier in the growing season enhances breeding decisions, thus accelerating the selection process. Lowering the cost of collecting trait data from more plots in diverse locations allows for more extensive evaluations. This increases the accuracy of genetic yield potential estimates and accelerates genetic gain per breeding cycle.

Over time, a variety of methods have been developed for yield forecasting using satellite data. Many studies combine satellite data with crop growth model estimations (de Wit and van Diepen, 2008; Jeong et al., 2018; Zhao et al., 2020; Zare et al., 2022; Luo et al., 2023), incorporate additional environmental factors like weather conditions (Bai et al., 2010; Schwalbert R. et al., 2020; Schwalbert RA. et al., 2020; Nieto et al., 2021; Lang et al., 2022), soil data (Broms et al., 2023; Mahalakshmi et al., 2025), and terrain information (Li et al., 2022; Sahbeni et al., 2023). Machine learning has been widely applied, using handcrafted features derived from raw images, including histogram-based representation of time-series data (You et al., 2017), and vegetation indices, particularly the Normalized Difference Vegetation Index (NDVI) and Enhanced Vegetation Index (EVI) (Yang et al., 2006; Yang et al., 2013; Johnson et al., 2016b; Peralta et al., 2016; Schwalbert et al., 2018; Cai et al., 2019; Mateo-Sanchis et al., 2019; Kamir et al., 2020; Khalil and Abdullaev, 2021; Meroni et al., 2021). While these indices have been effective, they are inherently limited by the scope of human expertise, restricting the ability to capture the full complexity of crop conditions (Feldmann et al., 2021).

Representation learning has emerged as a promising alternative approach for overcoming these limitations (Gage et al., 2019; Ubbens et al., 2020; Tross et al., 2023). Conventionally, various machine learning techniques, including Principal Component Analysis (PCA), Linear Discriminant Analysis (LDA), t-distributed Stochastic Neighbor Embedding (t-SNE), and autoencoders, have been applied to derive ‘latent representations’ from high-dimensional datasets (Zhong et al., 2016; Kopf and Claassen, 2021; Alexander et al., 2022; Gomari et al., 2022; Iwasaki et al., 2023; Song et al., 2023). Autoencoders stand out for their ability to identify non-linear patterns. Through the encoding of data into a reduced latent space followed by reconstruction of the input, these models generate a concise and meaningful representation that plays a key role in phenotyping (Gage et al., 2019; Ubbens et al., 2020; Tross et al., 2023). Despite their value, representations produced by autoencoders commonly struggle to differentiate between genetic and environmental effects, creating ‘entangled’ latent spaces in which specific plant traits—like ‘leaf number,’ ‘height,’ and ‘chlorophyll concentration’—are blended rather than distinctly isolated. Separating these traits in the latent space could enhance the interpretability of the resulting latent factors. Unlike traditional feature engineering, representation learning can automatically extract complex, high-dimensional features from raw input data without predefined formulas, potentially providing a richer understanding of crop traits and often outperforming traditional indices (Okada et al., 2024). This ability makes representation learning well-suited for capturing characteristics that influence crop performance. Recent studies have applied representation learning techniques to satellite data for various agricultural applications, such as classifying crop traits, mapping floods, and monitoring land use (Dumeur et al., 2024; Goyal et al., 2024; Nakayama and Su, 2024). While these methods have demonstrated success in large-scale yield estimation, they have yet to be broadly adopted for localized, plot-level predictions, where high precision is crucial for actionable insights.

Additionally, most existing approaches have not adequately addressed the complex interactions between genotype (G) and environment (E)— commonly referred to as GxE interactions — in their forecasting models. Incorporating GxE interactions is particularly important, as these interactions are a major source of variability in crop performance and the primary reason that the top-performing hybrids in one environment will often rank lower in relative performance in a second environment (Kusmec et al., 2018). A more nuanced understanding of how specific genotypes respond to different environmental conditions could significantly enhance prediction precision, especially at smaller spatial scales (e.g., plot-level).

**Figure 1:**
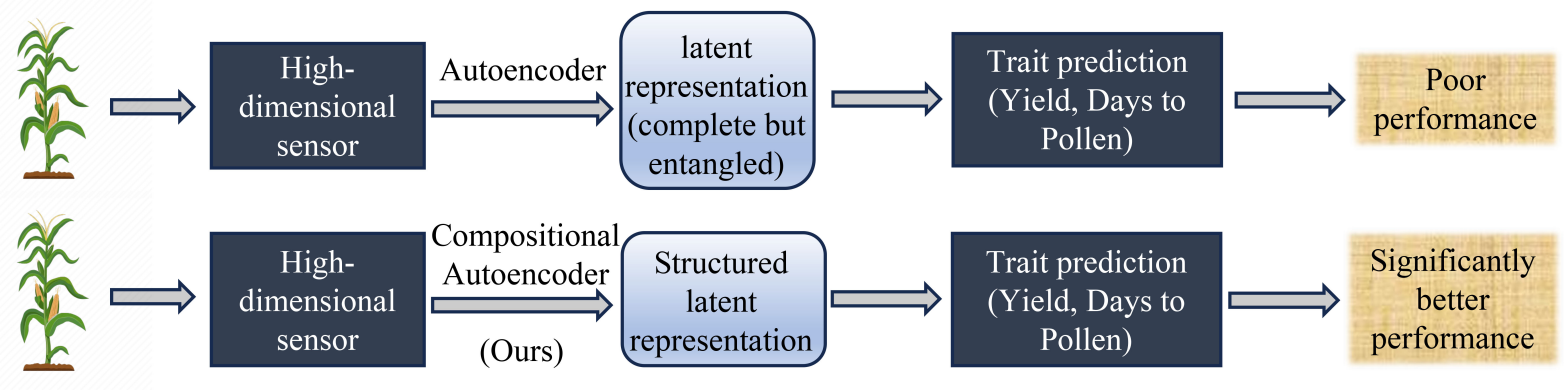
Overview of trait prediction from high-dimensional plant-level hyperspectral sensor data workflow and performance of Compositional Autoencoder (CAE) representation with other representation learning as shown in (Powadi et al., 2024).

In our recent work, we introduced a model, the Compositional Autoencoder (CAE), specifically designed to address these challenges (Powadi et al., 2024). The CAE integrates GxE interactions into the yield prediction framework, allowing for a more comprehensive representation of high-dimensional hyperspectral ground-based sensor data as GxE components (**Figure 1**). By capturing both genetic and environmental influences, the CAE outperformed traditional methods, demonstrating its potential as a valuable tool for improving the accuracy of yield predictions.

**Figure 2:**
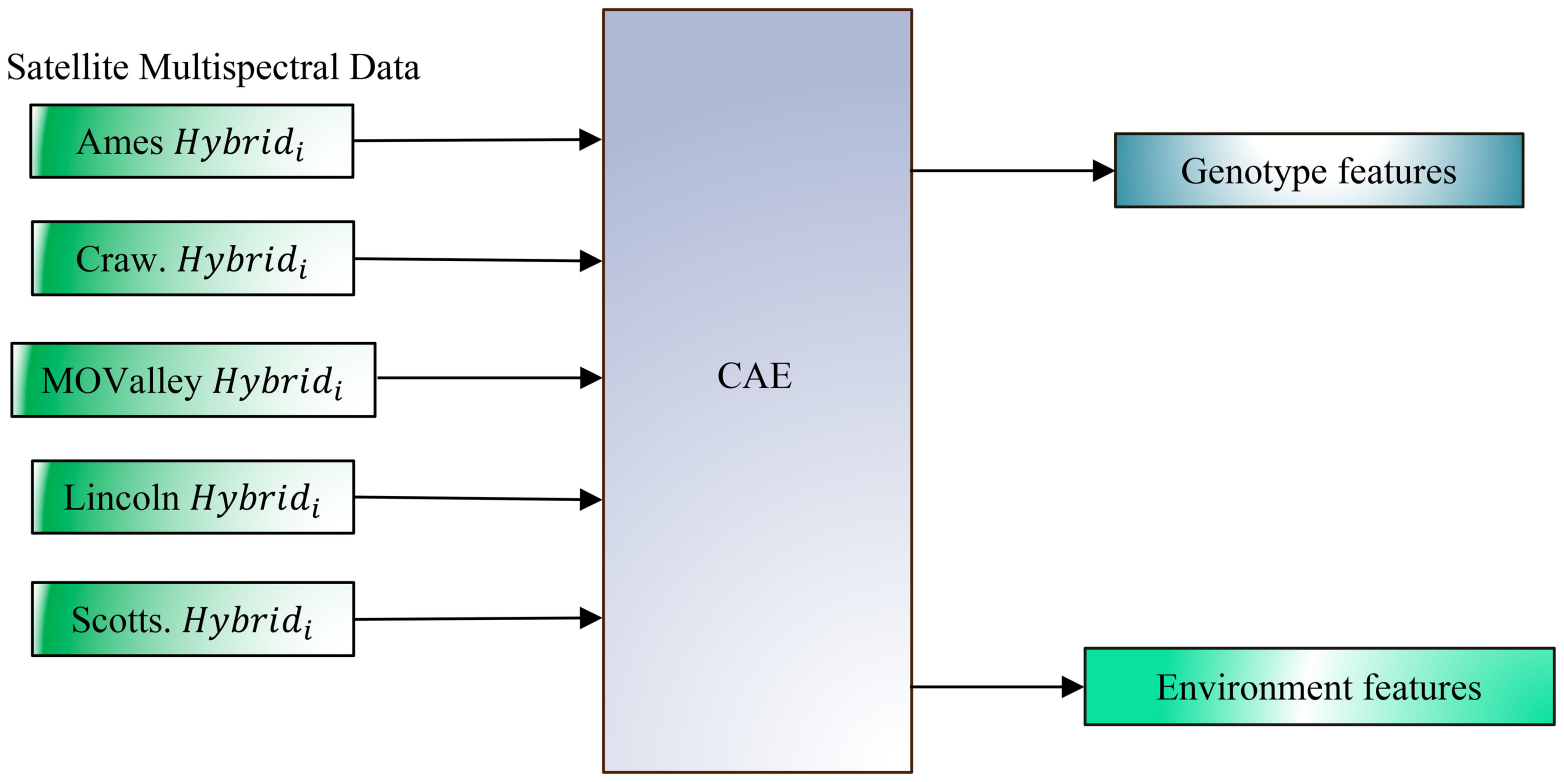
Disentangling genotype and environmental effects from plot-level multispectral canopy reflectance data, collected via satellite at a 30 cm resolution, across multiple environments and genotypes using a Compositional Autoencoder (CAE) framework. The figure illustrates data input from various locations (Ames, Crawfordsville (Craw), Missouri Valley (MOValley), Lincoln, and Scottsbluff (Scotts)), each with a specific genotype (*Hybrid_i_*) represented in its unique environmental context. The CAE (Compositional Autoencoder) framework converts this high-resolution data to separate genotype-specific and environment-specific features. By isolating these components, the model aims to improve the accuracy of yield prediction by capturing the independent contributions of both genotype and environment.

Building on this foundation, the present study aims to extend the CAE framework to satellite data at the plot level. This extension enables more precise yield forecasting and allows for deeper insights into the interactions between genetic and environmental factors. Our objectives in this work are to (a) evaluate CAE performance on disentangling genotype and environment features from satellite imagery collected at the plot scale to better understand their contributions to yield (**Figure 2**), and (b) to improve yield prediction accuracy by leveraging these disentangled features, as evidenced by the CAE’s superior performance over other representations, including traditional vegetation indices.

## 2 Materials and methods

### 2.1 Satellite images and yield data

#### 2.1.1 Locations and plants

Maize (*Zea mays L.*) field experiments were conducted at five locations: Scottsbluff, NE (41.85°N, -103.70°W), Lincoln, NE (40.86°N, -96.61°W), Missouri Valley, IA (41.67°N, -95.94°W), Ames, IA (42.01°N, -93.73°W), and Crawfordsville, IA (41.19°N, -91.48°W) in 2022. A map of these locations is provided in the **Supplementary Figure S1**. Depending on local weather conditions, planting occurred between April 29 and May 23, 2022. This work utilizes 84 hybrid genotypes planted in two replicated plots at each of these five locations.

The maize plants were cultivated under rain-fed conditions at four locations: Lincoln, Missouri Valley, Ames, and Crawfordsville. In contrast, the Scottsbluff site was irrigated, receiving a total of 16.9 inches (429.26 mm) of water over the growing season. All locations except Missouri Valley had one rate of nitrogen fertilization treatment of 150 lbs/acre. Nitrogen fertilization treatment for Missouri Valley was 175 lbs/acre. More details on the experimental design and data collection are available in Shresta et al (Shrestha et al., 2025). For one of the evaluation experiments of the CAE (experiment 3 in section 3.2), we additionally used satellite data of 45 common hybrids from Ames, Lincoln, and Missouri Valley from 2023.

#### 2.1.2 Satellite imagery and plot extraction

For this study, we utilized the Pléiades Neo satellite constellation to capture images at four different time points (TP), of which two were early in the season (July), referred to as TP1 and TP2, capturing the late vegetative stage and early flowering stages. Two others were late in the season (September), referred to as TP3 and TP4, capturing mature and harvest-ready plants. The growth-stage timeline for the maize plants was recorded for the plants grown in Lincoln is shown in the **Supplementary Figure S2**. **Table 1** provides the specifications of the Pléiades Neo satellite constellation used in our study. The six bands in the satellite multispectral images cover the following spectral ranges: Red (620–690 nm), Green (530–590 nm), Blue (450–520 nm), Near-infrared (NIR, 770–880 nm), Red Edge (700–750 nm), Deep Blue (400–450 nm).

**Table 1:** Satellite specifications for the Pléiades Neo satellite constellation used in our study.

Parameter	Description
Orbit	Sun-synchronous, 10:30 a.m. descending node, 620 km altitude
Number of Satellites	2 identical satellites in constellation
Sensor Bands	Panchromatic (450–800 nm)
	Multispectral: • Red (620–690 nm) • Green (530–590 nm) • Blue (450–520 nm) • Near-infrared (770–880 nm) • Red Edge (700–750 nm) • Deep Blue (400–450 nm)
Sensor Resolution	30 cm (panchromatic band), 1.2 m (multi-spectral bands)
Dynamic Range	12-bits per pixel
Swath Width	At nadir: 14 km
Revisit Frequency	Daily anywhere (30° off-nadir) or twice daily anywhere (45° off-nadir)
Acquisition Capacity	Up to 2 million *km* ^2^ per day

**Figure 3:**
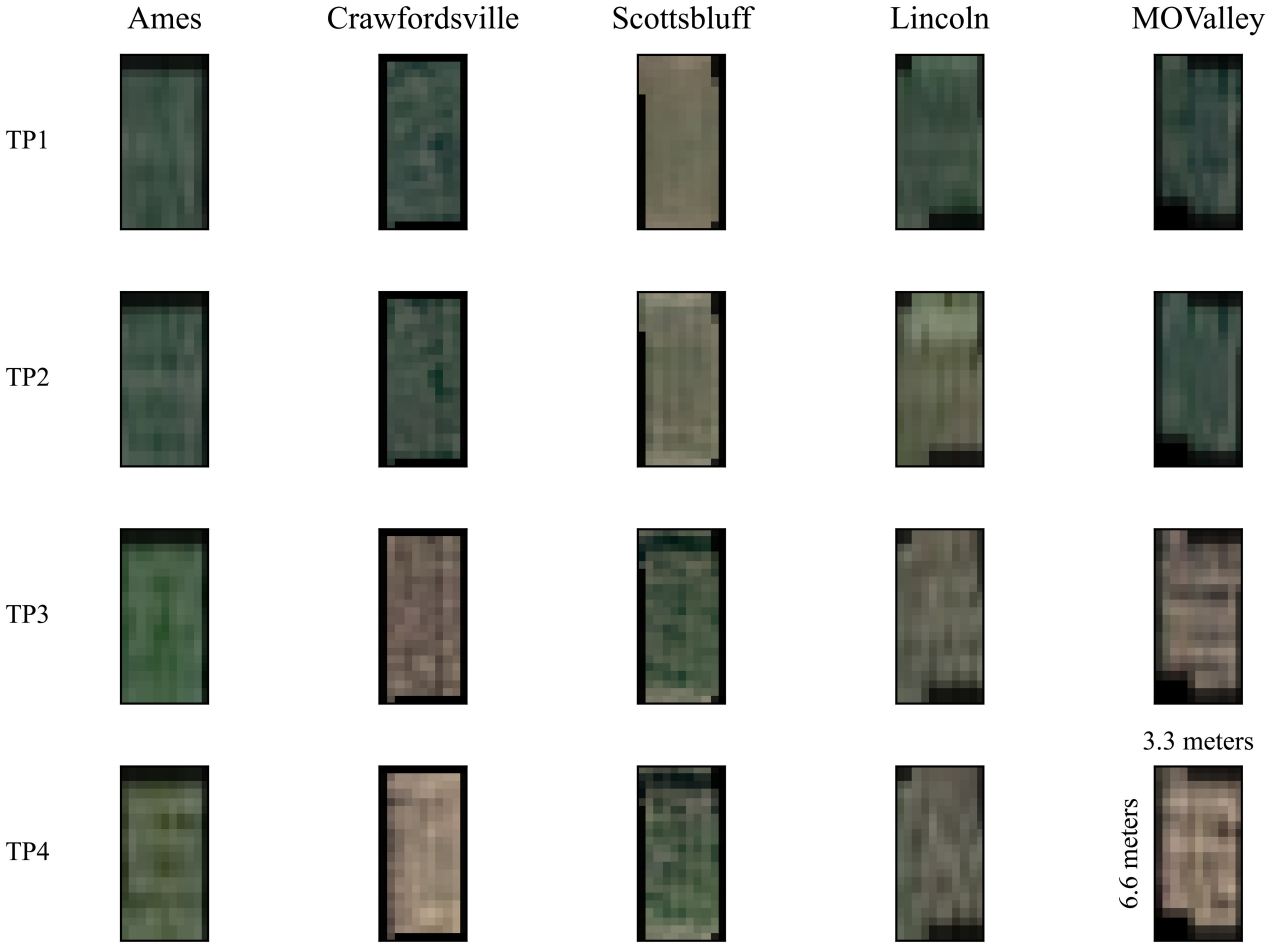
Satellite data samples from an arbitrary plot at each location are shown across four timepoints (TP), with the first two collected early in the growing season (July) and the last two collected toward the end of the season (September). Only the R, G, and B channels are visualized. Each plot is 11 by 22 pixels, representing 3.3 meters by 6.6 meters (as shown in TP4, MOValley). The distinctive variations among images at each time point are primarily due to differences in planting dates, which ranged from April 29 to May 23, 2022, depending on local weather conditions. Notably, images from Scottsbluff exhibit visible differences, as they were captured from a field planted later than the others.

In addition to multispectral images, a single-band panchromatic raster file with a wide bandwidth of approximately 450–800 nm was generated. Each image covered a total area of 100 km × 100 km per location, encompassing the entire experimental field at each location. The final 16-bit GeoTIFF satellite images with 30-cm resolution were generated after panchromatic sharpening (pansharpening), manual orthorectification, and atmospheric correction by the service provider prior to the delivery of imagery. The plot-level images were extracted from the satellite data as described in our previous paper (Shrestha et al., 2025). The extracted plot images are of the shape 11 x 22 x 6 (Width x Height x Channels) pixels. **Figure 3** shows samples of satellite image data for the 4 time points for all the locations we capture. The plots measure approximately 3.3 meters by 6.6 meters (shown in **Figure 3** on the TP4 sample for the Mo Valley), covering an area of 21.78 square meters. With the Pléiades Neo satellite’s native spatial resolution of 30 cm (0.3 meters) per pixel, each pixel represents a ground area of 0.09 square meters. A single plot would thus be sampled by approximately 11 pixels along the width and 22 pixels along the length, for a total of about 242 pixels. This limited pixel count per plot results in the expected pixelated appearance in the imagery, as fine details smaller than the resolution cannot be resolved. The plot boundaries and extraction were performed using the ArcPy Jupyter Notebook environment implemented in ArcGIS Pro V3.2.0.

#### 2.1.3 Preprocessing of plot images

**Figure 4:**
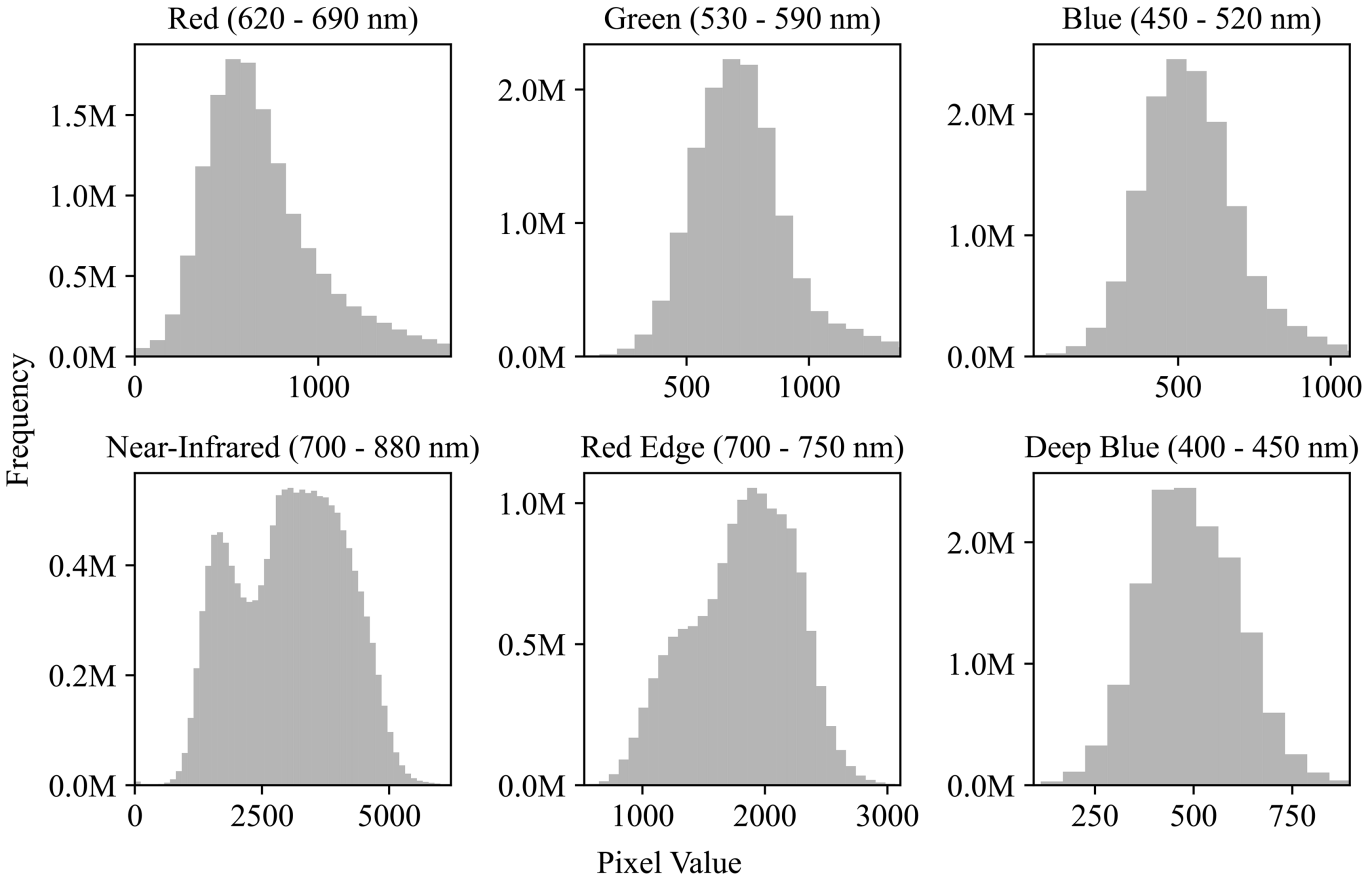
Pixel value distributions across spectral channels. Histograms show the frequency of pixel values for six satellite image channels: Red (620–690 nm), Green (530–590 nm), Blue (450–520 nm), Near-Infrared (700–880 nm), Red Edge (700–750 nm), and Deep Blue (400–450 nm). Although the image data is 16-bit (range: 0−65,536), most pixel values fall within 0−5000, indicating a limited dynamic range in practice.

The preprocessing of the plot images was divided into two stages: outlier removal and normalization. For each spectral channel, outliers were removed by clipping values outside 3 standard deviations from the mean. **Figure 4** illustrates the distribution of pixel values across channels after outlier removal. The 6-channel data were then normalized on a per-channel basis using min-max scaling. After normalization, each plot image was flattened into a 1 x 1452 vector before being fed into the neural network.

#### 2.1.4 Yield data

Yield data is recorded at the time of the harvest in the field. Plants were mechanically harvested from the two middle rows of each four-row plot using a plot combine. At the time of harvesting, the combine recorded percent moisture content, on a fresh weight basis, and the fresh weight of the grain in pounds. To compare across locations, yield in bushels per acre at 15.5% moisture on a fresh weight basis and 56 pounds per bushel was calculated based on these values and the total area occupied by the middle two rows of each plot at each location.

**Figure 5:**
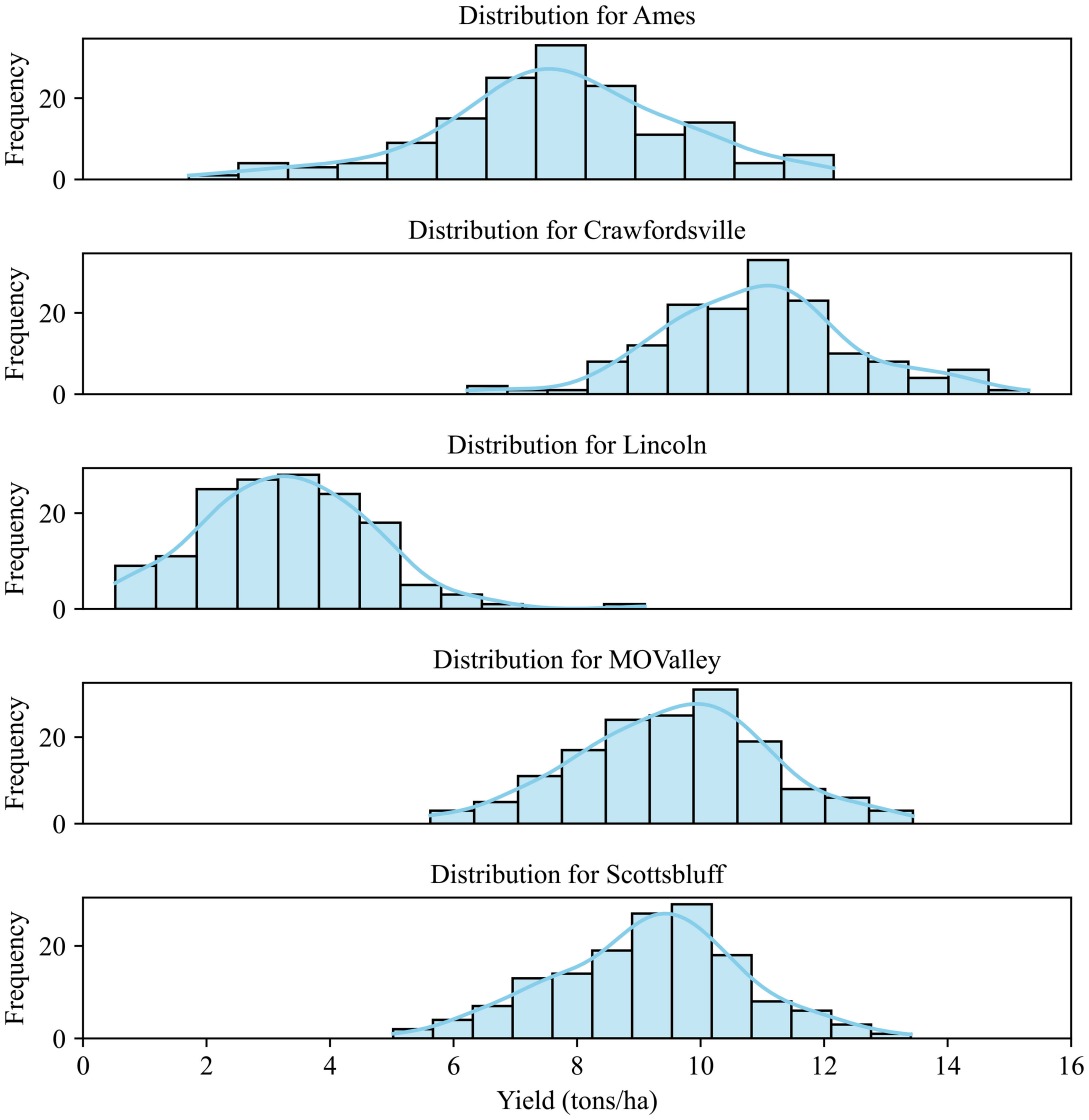
Yield distribution for the 84 hybrids across different environments (locations). The plants were grown under rain-fed conditions in Lincoln, Missouri Valley, Ames, and Crawfordsville. The experimental field in Scottsbluff was irrigated.

The yield data were recorded for these 84 hybrids, which were used for evaluating the predictive capabilities of the CAE as well as the degree of disentanglement of environmental features. **Figure 5** shows the yield distribution for different locations. As can be expected, genotypes respond differently to different environments. Clearly, Crawfordsville shows the highest yield overall, while Lincoln shows the lowest yield distribution. While different yields in different environments are expected due to the differences in soil, weather, and elevation conditions, Lincoln produced the lowest yield due to intense weed pressure. A deeper analysis of these yields and experiments can be found in the previous work (Shrestha et al., 2025).

### 2.2 Autoencoder

**Figure 6:**
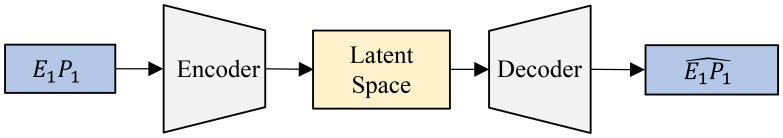
An autoencoder processes a single image (for example, *E*1*P*1 (Environment 1 - Replicate 1)) through an encoder to compress it into a lower-dimensional latent space, capturing its essential features. The decoder then reconstructs the input from this compressed representation.

An autoencoder is a type of neural network designed for representation learning, enabling the extraction of efficient and compressed representations of input data. It consists of two main components: an encoder and a decoder. The encoder compresses the input data into a latent representation, while the decoder aims to reconstruct the original input from this latent space. Through this process, the autoencoder effectively captures the essential characteristics of the input data, thereby learning meaningful representations. **Figure 6** shows our implementation, where the encoder and decoder are built using multiple fully connected layers, each utilizing the SeLU activation function. The encoder compresses the 6-channel satellite input data into a lower-dimensional latent space, which retains key features of the input while being highly informative. The decoder then reconstructs the original input from this compressed latent representation. **Tables 2a, b** provide detailed information about the specific layers used in the encoder and decoder. This encoder-decoder architecture forms a latent space that is both compact and representative of the input data, providing added benefits of noise reduction and feature enhancement. This “vanilla autoencoder” serves as our baseline representation learning model.

**Table 2:** Configuration details for encoder and decoder.

Layer type	Dimensions	Activation
(a) Encoder Configuration		
Linear	input shape → 2200	SELU
Linear	2200 → 2000	SELU
Linear	2000 → 1000	SELU
Linear	1000 → zg + ze + zp	None
(b) Decoder Configuration		
Linear	zg + ze + zp → 2024	SELU
Linear	2024 → 3000	SELU
Linear	3000 → 2200	SELU
Linear	2200 → input_shape	Sigmoid

input shape = 1 x 1452, zg = dimensions for genotype features, ze = dimensions for macro-environment features, zp = dimensions for microenvironment features.

### 2.3 Compositional autoencoder

The Compositional Autoencoder (CAE) builds upon the standard autoencoder to disentangle the latent space into multiple components that reflect various influential factors in the data. By learning structured representations, the CAE captures general, environmental, and specific attributes, making it significantly more informative than a conventional autoencoder. The CAE is composed of three primary components: an encoder, a fusion block, and a decoder.

#### 2.3.1 Architecture overview

The CAE architecture retains the traditional encoder-decoder structure, with the addition of a fusion block to combine and disentangle encoded data from multiple images. Satellite data of the same genotype across all the locations are grouped together in 84 groups (84 being the number of genotypes). Each group will have 5 images (for 5 locations), which will be sequentially passed to the encoder, resulting in a separate latent representation for each image in the group. The encoder used here is identical to the one implemented in the standard autoencoder, ensuring consistency in feature extraction across both approaches.

The latent features derived from these images are then aggregated using a fusion layer. This fusion layer operates by combining the encoded information, resulting in a unified latent vector that integrates features from all input images. A detailed description of this fusion mechanism is provided in **Table 3**.

**Table 3:** Fusion layer details.

Layer type	Dimensions	Activation
Linear	N(zg + ze + zp) → zg + E(ze) + N(zp)	None

‘N’ = number of replicates per image, ‘E’ = number of environments, ‘zg’ = dimensions allocated to capture general features, ‘ze’ = dimensions allocated to capture environmental features, ‘zp’ = dimensions allocated to specific features.

Once fused, the latent representation is divided into three distinct components. The general features capture shared characteristics present in all images. The environmental features represent attributes specific to a given environment, shared across images from similar conditions. Finally, specific features represent unique aspects that differ across each image, such as particular objects or anomalies. This structured separation ensures that the CAE can provide a richer, more nuanced representation of the input data.

**Figure 7:**
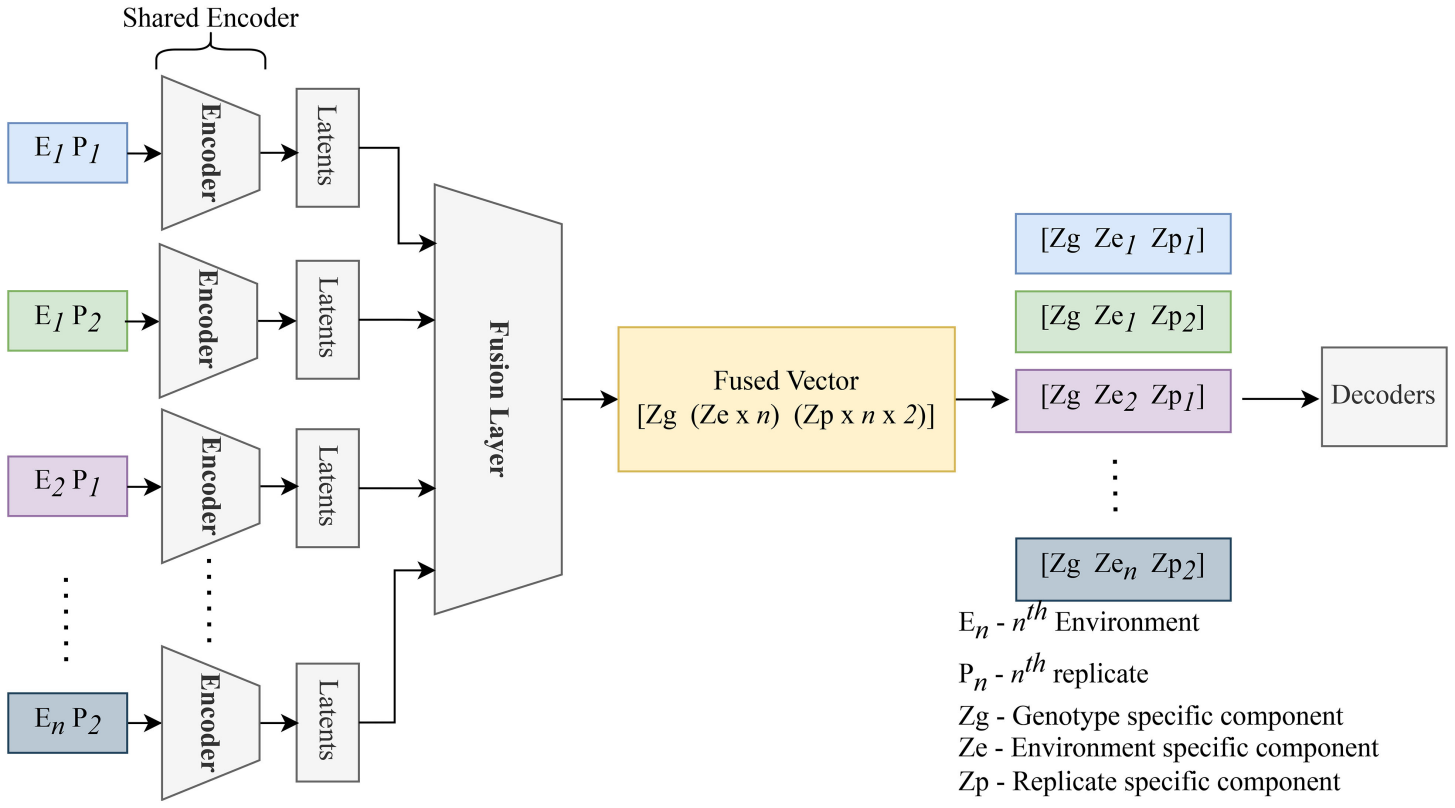
Replicates of the same genotype are grouped together and fed into the CAE during the forward pass. *E*_1_ *P*_1_ represents environment replicate 1. All these replicates are fed into the encoder sequentially to generate latents as shown above. These latent spaces are then fused together using a fusion layer. We enforce the partitions in this fusion vector (Genotype (Zg), macroenvironment response (Ze), and micro-environment-response (Zp)). Each macro-environment and micro-environment will have its own partition. Next, Partitions are picked for each replicate and unique representation for each replicate is obtained. These latent representations are then sequentially given to the decoder to reconstruct the input.

After this separation, the decoder reconstructs each original image by reassembling the general, environmental, and specific features. The decoder, identical to that used in the basic autoencoder, leverages the latent space to reconstruct the input, thereby enabling an in-depth understanding of the underlying factors driving variations in the satellite data. **Figure 7** presents an overview of the CAE architecture, showing how different components contribute to reconstructing the input images. The detailed structure of the fusion layer is provided in **Table 3**, and **Table 4** demonstrates the disentangled latent representation for each individual image.

**Table 4:** Disentangled latent-space representation of each image.

Image	Representation
*E*_1_ *P*_1_	{(Zg) genotype features, (Ze) macro-env. features [1], (Zp) micro-env. features [1]}
*E*_1_ *P*_2_	{(Zg) genotype features, (Ze) macro-env. features [1], (Zp) micro-env. features [2]}
*E*_2_ *P*_1_	{(Zg) genotype features, (Ze) macro-env. features [2], (Zp) micro-env. features [3]}
*E*_2_ *P*_2_	{(Zg) genotype features, (Ze) macro-env. features [2], (Zp) micro-env. features [4]}
*E*_3_ *P*_1_	{(Zg) genotype features, (Ze) macro-env. features [3], (Zp) micro-env. features [5]}
*E*_3_ *P*_2_	{(Zg) genotype features, (Ze) macro-env. features [3], (Zp) micro-env. features [6]}
*E*_4_ *P*_1_	{(Zg) genotype features, (Ze) macro-env. features [4], (Zp) micro-env. features [7]}
*E*_4_ *P*_2_	{(Zg) genotype features, (Ze) macro-env. features [4], (Zp) micro-env. features [8]}
*E*_5_ *P*_1_	{(Zg) genotype features, (Ze) macro-env. features [5], (Zp) micro-env. features [9]}
*E*_5_ *P*_2_	{(Zg) genotype features, (Ze) macro-env. features [5], (Zp) micro-env. features [10]}

#### 2.3.2 Loss function

To effectively train the CAE, a two-part loss function is used, consisting of a reconstruction loss and a correlation loss.

The reconstruction loss, computed as the mean squared error (MSE), ensures that the network can accurately reconstruct the original satellite images from the learned latent space. This loss encourages the CAE to capture the most relevant features for high-quality reconstruction, ensuring that the latent space is both informative and efficient.

The correlation loss is applied to maintain the independence of the disentangled components — general, environmental, and specific features — within the latent space. This loss penalizes any correlation between different parts of the latent space, thus enforcing their distinctiveness and improving the interpretability of the model. The correlation loss is expressed as:


(1) $$\text{Correlation Loss}=\sum_{i=1}^{N}\sum_{j=i}^{N}|{\text{CorrMat}}_{ij}|-I_{ij}$$

In this equation, CorrMat*_ij_* represents the correlation coefficient between the latent space dimensions *i* and *j*, and *N* denotes the size of the square correlation matrix. The term *I_ij_* refers to the identity matrix, which ensures that the diagonal elements (where *i* = *j*) do not contribute to the loss, thus emphasizing only the off-diagonal correlations.

The combination of reconstruction and correlation loss allows CAE to learn latent representations that are informative, distinct, and suitable for downstream tasks, such as yield prediction and analysis of genotype-by-environment interactions. The correlation coefficient used here is the Pearson correlation coefficient (*r*), which measures the linear relationship between two variables. It is defined by the following equation:


(2) $$r=\frac{\sum_{i=1}^{n}\left(p_{i}-\bar{p})(k_{i}-\bar{k}\right)}{\sqrt{\sum_{i=1}^{n}{\left(p_{i}-\bar{p}\right)}^{2}\sum_{i=1}^{n}(k_{i}-\bar{k}{)}^{2}}}$$

In this equation (Equation 2), *n* represents the number of data points. $p_{i}$ and $k_{i}$ are elements from different dimensions of the latent space. $\bar{p}$ and $\bar{k}$ are the means of the $p^{th}$ and $k^{th}$ dimensions, respectively. Our objective is to achieve zero correlation between the latent space features that represent general, environmental, and specific variations. By enforcing this condition through the correlation loss function (Equation 1), the model ensures that each disentangled component captures its respective factor independently, leading to a more interpretable and effective representation of the underlying data.

## 3 Results and discussion

### 3.1 Environment disentanglement

We first assess the degree of disentanglement achieved by the Compositional Autoencoder (CAE) across different environments in our dataset.

**Figure 8:**
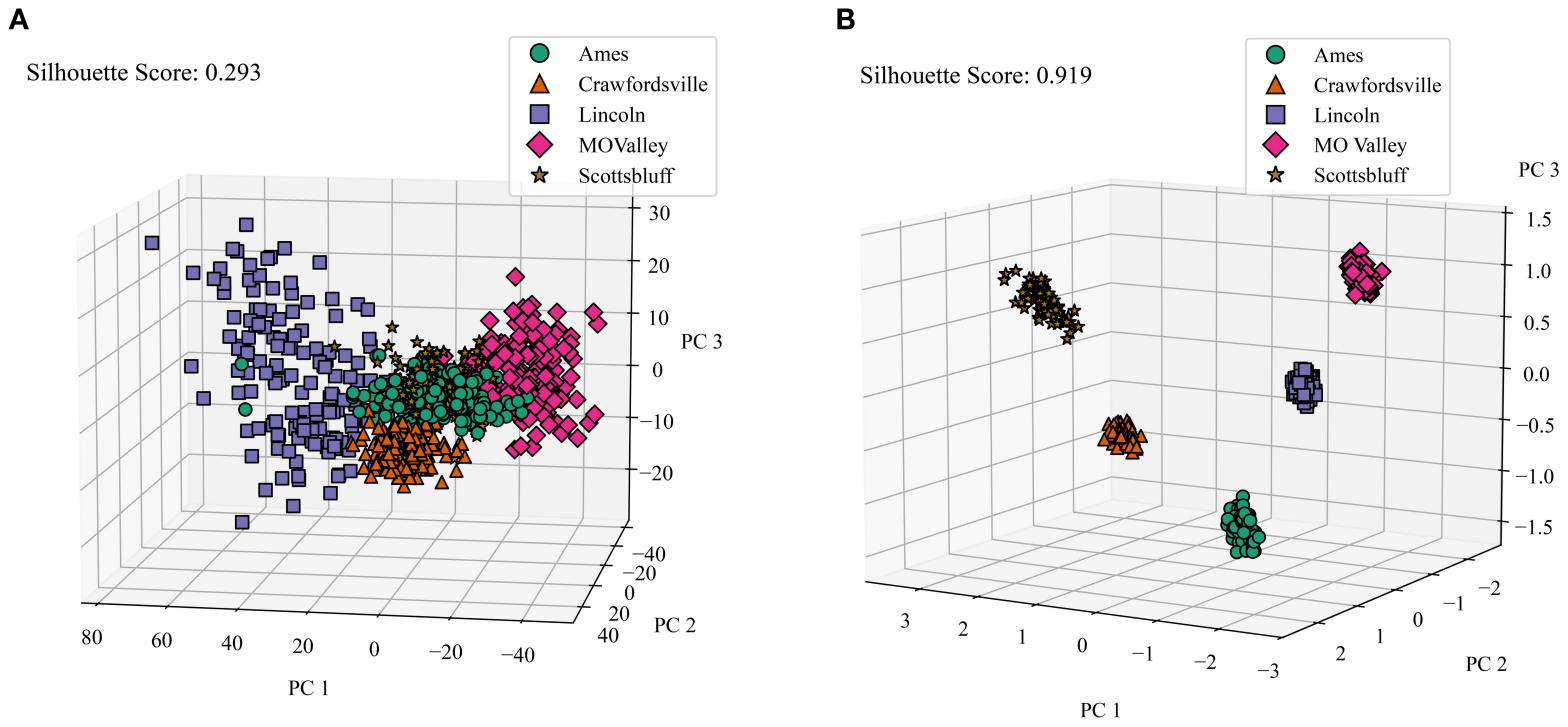
Comparison of Raw Data Clusters and Macro Environment Disentanglement. We applied PCA on the raw reflectance data and latent features generated by the CAE (we averaged the genotype and micro-environment components), and color-coded the features from different locations and calculated the silhouette score to determine how well all of the clusters are separated. **(A)** Macro environment clusters visualized for raw data. PC 1, PC 2, and PC 3 are the top principal components 1, 2, and 3, respectively. Cumulative variance explained = 0.695. Variance explained: PC 1 = 0.4, PC 2 = 0.24, PC = 0.04), **(B)** Macro Environment Disentanglement Visualized. Cumulative variance explained = 0.94. Variance explained: PC 1 = 0.474, PC 2 = 0.268, PC 3 = 0.201.

Our dataset comprises information from five distinct locations. To establish a baseline, we first analyzed how well the raw satellite data differentiates among these locations. We applied Principal Component Analysis (PCA) to the raw data, selecting the first three principal components. These components were then visualized in a 3D plot, with data points color-coded by location. To quantify the distinctness of the resulting clusters, we calculated their silhouette scores. The silhouette score measures how well data points in a cluster are grouped by comparing the average intra-cluster distance (how close points are within the same cluster) to the nearest inter-cluster distance (how far they are from points in the nearest cluster). Scores range from -1 to 1, where values close to 1 indicate well-separated, compact clusters, and values near or below 0 suggest overlapping or poorly defined clusters. **Figure 8A** illustrates this visualization of the raw data. Notably, the clusters representing different locations show significant overlap, resulting in a low silhouette score of only 0.293. This indicates that the raw satellite data alone does not effectively distinguish among environments.

To evaluate the environmental disentanglement achieved by the CAE, we analyzed its latent space. The CAE’s latent space is divided into ‘genotype’, ‘macro-environment’, and ‘micro-environment’ components. For this analysis, we consider the ‘macro-environment’ component (and average over the ‘genotype’ and ‘micro-environment’ components) and perform the same clustering and visualization process as with the raw data. **Figure 8B** displays the clusters for all five locations generated by the CAE. Strikingly, the silhouette score for these clusters is 0.919, a substantial improvement over the raw data. This high score indicates that the CAE has successfully disentangled macroenvironmental factors, creating much more distinct clusters for each location.

**Figure 9:**
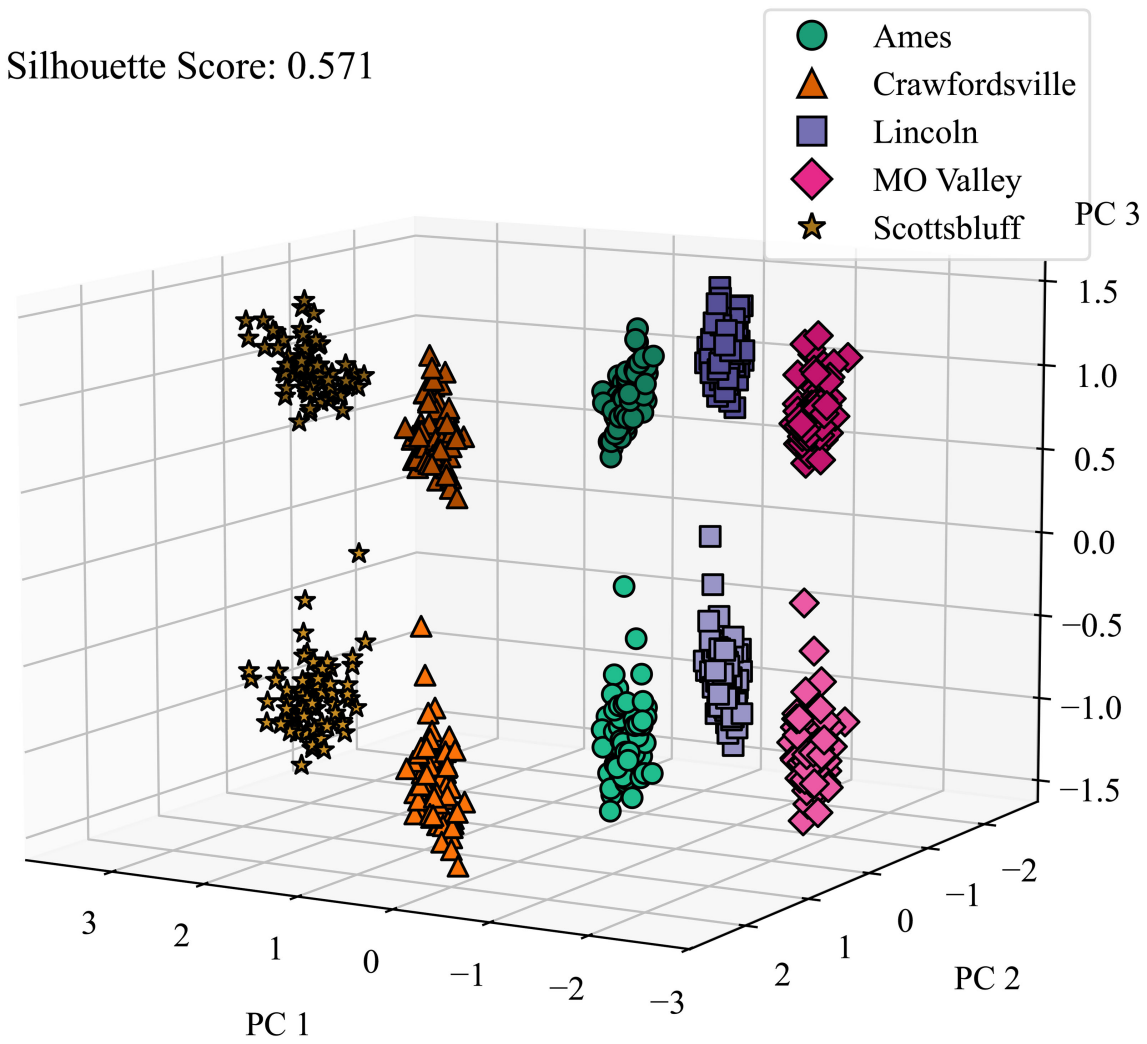
Micro environment disentanglement visualized. We applied PCA on latent features generated by the CAE and color-coded the features from different locations and calculated the silhouette score to determine how well all of the clusters are separated. Micro-environment refers to the local environment around a plot. Lighter and darker shades of the same color represent the microenvironments around 2 replicates grown in the same macro-environment. PC 1, PC 2, and PC 3 are the top principal components 1, 2, and 3, respectively.

We also investigated the CAE’s ability to disentangle micro-environmental factors. As shown in **Figure 9**, the CAE demonstrates considerable success in this aspect as well, further highlighting its robustness in environmental disentanglement. These results suggest the CAE’s potential to separate environmental factors detected via satellite imagery, which could lead to a more nuanced understanding of how different environments may contribute to plant characteristics and performance.

### 3.2 Performance on yield prediction

We next conducted multiple experiments to assess the performance of the latent vectors constructed by the Compositional Autoencoder (CAE) in predicting maize yield. Each experiment consists of using the latent representation as input to train a machine-learning model that predicts maize yield in the plots. The latent configuration used for all these downstream predictions shown in this section is ‘6-6-1’ (zg-ze-zp) which was selected based on the sensitivity analysis provided in **Supplementary Table S2**. For yield prediction, we used the XGBoost model, which consistently outperformed other machine learning algorithms in our preliminary evaluation. Comparative results with classical models such as PLSR, Ridge regression, and Random Forest are provided in **Supplementary Tables S3** and **S4**. The hyperparameter settings used for each model configuration are summarized in **Supplementary Tables S2**. The three experiments were designed to evaluate model performance across different generalization scenarios. The corresponding data splits and evaluation protocols are described in **Supplementary Section 6**. In experiment 1, we compare the predictive capability of the CAE latent vectors against two baselines: latent vectors generated by a vanilla autoencoder (AE) and vegetation indices (VIs). We perform cross-validation across genotypes. In experiment 2, we evaluate the ability of the CAE-based latent vectors to rank order genotypes according to their predicted yield. Early and accurate rank ordering is a critical functionality in plant breeding, where early and accurate ranking can guide selection decisions. Finally, in experiment 3, we evaluate the performance of the latent CAE representation to predict the yield for a set of genotypes in an unseen environment (using data collected in Ames in the next year, 2023).

#### 3.2.1 Experiment 1: CAE vs AE vs VIs yield prediction comparison, with 5-fold crossvalidation

**Figure 10:**
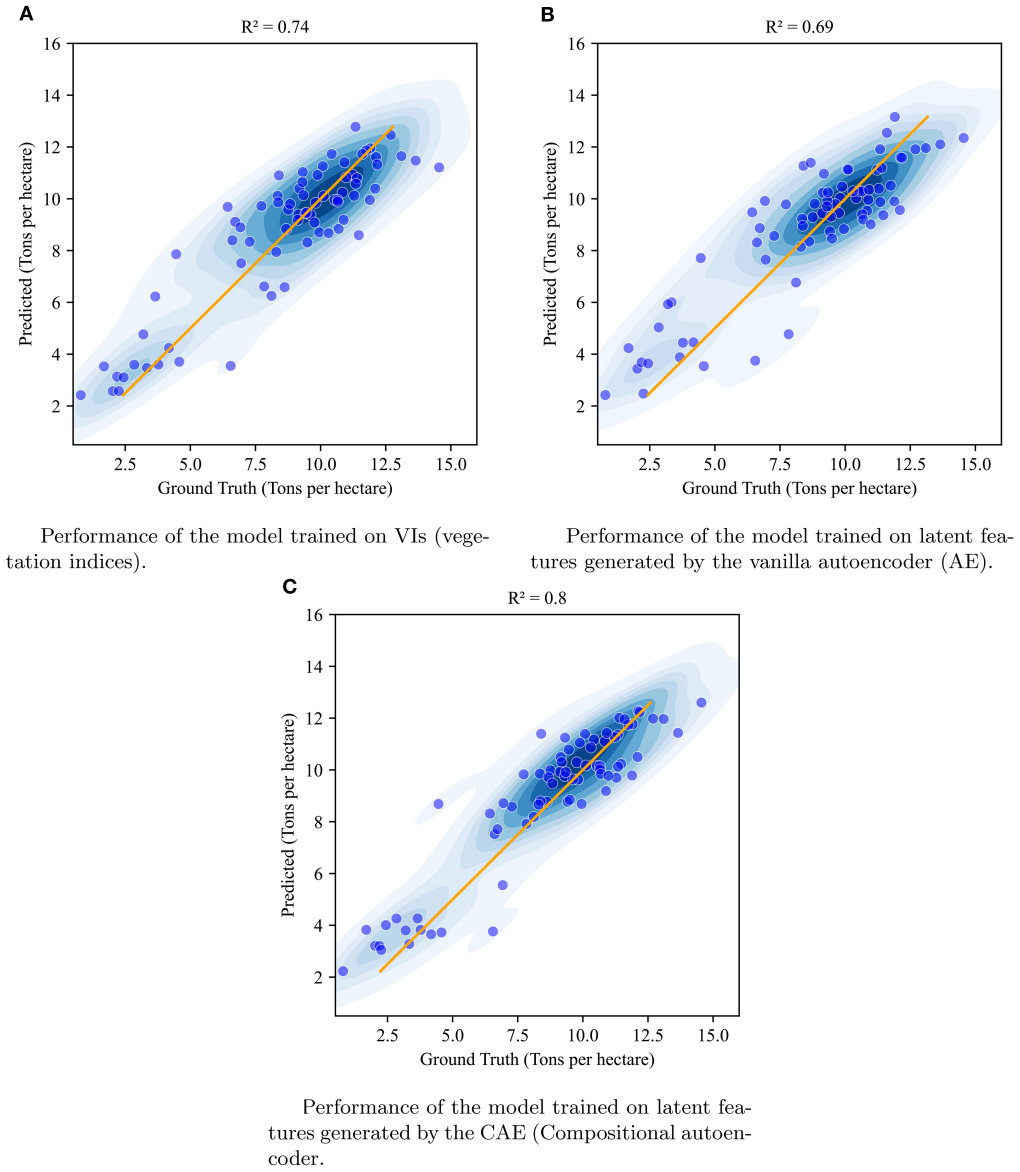
Visualization of the performance of XgBoost model when using vegetation indices **(A)**, vanilla autoencoder generated latent features **(B)**, and compositional autoencoder generated latent features **(C)** at timepoint 1.

We compared the yield prediction performance of the CAE latent vectors with a vanilla autoencoder (AE) latent vectors and vegetation indices (VIs) using 5-fold cross-validation across four key time points. **Table 5** presents a summary of the results, including R^2^ values and Root Mean Square Error (RMSE) in tons per hectare, which offer robust estimates of model performance across different timepoints. A visualization of these fits is given in **Figure 10** with absolute values of the yield (tons/ha) for timepoint 1 (TP1). As the k-folds were grouped based on genotypes, the results here reflect the accuracy with which the performance of unseen genotypes could be predicted in observed environments.

**Table 5:** Prediction performance of CAE (compositional autoencoder), AE (vanilla autoencoder), and VIs (vegetation indices) across timepoints 1, 2, 3, and 4, covering both early growth stages and near-harvest stages of the growing season.

Model	Timepoint	R2	RMSE (tons/ha)
CAE	1	**0.79 (0.05)**	**1.36 (0.16)**
AE	1	0.67 (0.04)	1.69 (0.13)
VIs	1	0.75 (0.03)	1.48 (0.12)
CAE	2	0.79 (0.043)	1.34 (0.14)
AE	2	0.72 (0.057)	1.56 (0.16)
VIs	2	**0.80 (0.04)**	**1.30 (0.15)**
CAE	3	**0.77 (0.07)**	**1.41 (0.14)**
AE	3	0.27 (0.1)	2.51 (0.21)
VIs	3	**0.77 (0.07)**	**1.41 (0.20)**
CAE	4	**0.76 (0.04)**	**1.45 (0.14)**
AE	4	0.45 (0.06)	1.51 (0.12)
VIs	4	0.70 (0.07)	1.41 (0.18)

Results are reported for yield prediction. Each entry shows the mean value, with the standard deviation from 5-fold cross-validation in parentheses. Bold values indicate the best performance for a given timepoint. *R* ^2^ is a unitless indicator of model fit, whereas RMSE is expressed in yield units (tons/ha) and represents the absolute prediction error.

The results clearly demonstrate that the CAE latent vector based predictor consistently outperforms the AE latent vector based predictor across all growth stages. Notably, the CAE latent vector based predictor shows higher R^2^ values and lower RMSE compared to the AE latent vector based predictor at every time point. The performance difference is particularly evident at time point 3, where the CAE latent vector based model maintains a relatively strong performance (R^2^ of 0.766) while the AE’s performance drops significantly (R^2^ of 0.27). Overall, the CAE latent vector based predictor demonstrates more consistent and robust performance throughout the growing season. Additionally, when compared with a model that uses vegetation indices (VIs) (**Table 5**), the CAE latent vector based predictor consistently outperforms them at timepoints 1 and 4 and shows comparable performance at time points 2 and 3. This comparable performance during timepoint 2 can be attributed to the fact that plant growth at this stage is near its peak, which correlates well with final yield. The vegetation indices used in this paper are given in the **Table 6**. Their description is provided in the **Supplementary Materials**. The CAE latent vector based predictor demonstrates particular strength in early-season predictions, with improvements (over AE) of approximately 11% and 7% in R^2^ values for the first two time points, respectively. Moreover, the CAE latent vector based predictor maintains more consistent performance across all time points, illustrating its robustness throughout the growing season.

**Table 6:** Vegetation indices (VIs) used in this study, along with their full names and source references.

VI	Full form	Citation
GLI	Green Leaf Index	(Mounir Louhaichi and Johnson, 2001)
NGRDI	Normalized Green-Red Difference Index	(Tucker, 1979)
VARI	Visible Atmospherically Resistant Index	(Gitelson et al., 2002)
VEG	Vegetation Index	(Hague et al., 2006)
RGBVI	Red-Green-Blue Vegetation Index	(Bendig et al., 2015)
ExG	Excess Green Index	(Woebbecke D et al., 1995)
ExR	Excess Red Index	(Meyer and Neto, 2008)
NDVI	Normalized Difference Vegetation Index	(Rouse et al., 1974)
GNDVI	Green Normalized Difference Vegetation Index	(Gitelson et al., 1996)
EVI	Enhanced Vegetation Index	(Huete et al., 2002)
SAVI	Soil-Adjusted Vegetation Index	(Huete, 1988)
NDRE	Normalized Difference Red Edge	(Gitelson and Merzlyak, 1994)
RDVI	Renormalized Difference Vegetation Index	(Roujean and Breon, 1995)

The improved yield prediction accuracy demonstrated by the Compositional Autoencoder (CAE) latent vector based predictor suggests utility for both farmers and plant breeders. For example, in the early season, one month after planting, the CAE achieves an R^2^ of 0.785 compared to the traditional autoencoder’s 0.67, representing an 11% improvement and around 4% improvement over vegetation indices-driven predictions. This accuracy increases further at 1.5 months post-planting, with the CAE latent vector based predictor reaching an R^2^ of 0.793. In the late-season, the final timepoint, close to harvest, the CAE maintains a high R^2^ of 0.757, significantly outperforming the traditional autoencoder’s 0.45 and also those of vegetation indices by 6%. To statistically validate these performance gains, we conducted pairwise ANOVA tests across all timepoints. The results show statistically significant differences in model performance for time points 1 and 4, with CAE significantly outperforming the baseline methods (see **Supplementary Tables S5-S8**). Such early predictions could help growers make timely decisions about resource allocation, potentially improving crop management, resource use efficiency, and yield.

#### 3.2.2 Experiment 2: rank ordering of top yielding genotypes

**Figure 11:**
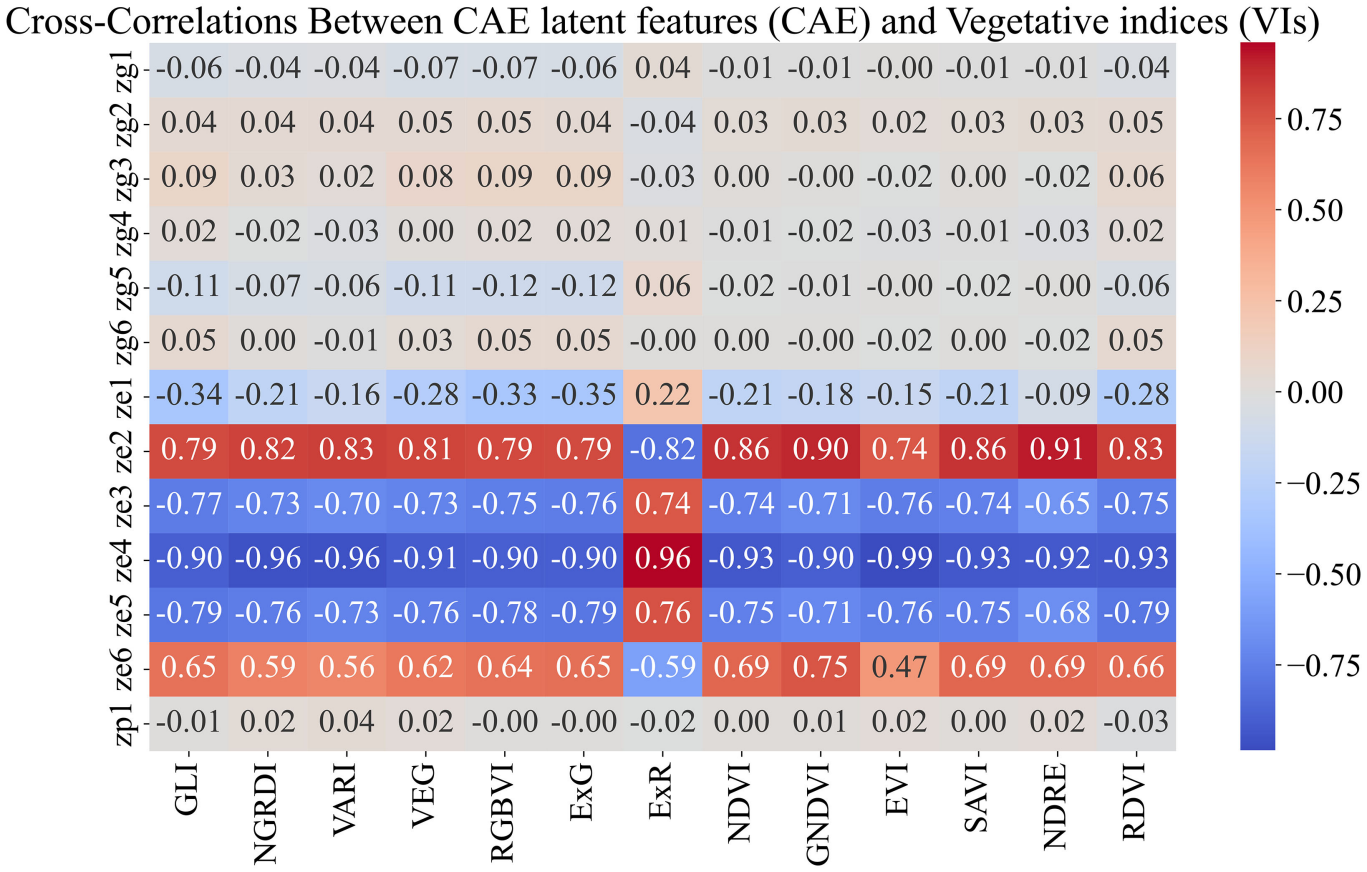
Feature correlations for combined feature space (CAE+VIs). We observe that the VIs show high correlation with the ‘ze’ components generated by CAE. High correlations between these feature sets will introduce redundant information, which could negatively affect the model’s performance.

Breeders often rank order varieties to make breeding selections. We evaluated the ability of the CAE latent vector-based predictor to rank order the top 25% and top 50% performing genotypes. We consider time-point 2. This is around the time all the maize plants are near the end of the vegetative stage. We trained the same XGBoost model as before (which uses the disentangled latents as inputs and the yield as outputs) and performed a leave-one-location-out evaluation. That is, for evaluating the performance of a location, we trained the model on all the other locations and tested the model on that location. For example, for evaluating ‘Ames’, we trained the model on the data from all the other locations and then tested its performance on that of ‘Ames’. To understand how well a model is able to capture the top n% highest-performing genotypes, we consider the list of top-yielding genotypes for each location and then calculate the percentage overlap of the top-yielding genotypes from the respective predictions. **Tables 7a, b** show the performance of CAE features and vegetation indices for the top 25% and top 50%. We observe that the model trained on CAE features consistently outperforms the model trained using vegetation indices, with one exception for the top 25% (Scottsbluff). We can also see that using both of these feature sets together does not seem to improve the performance for most cases. To better understand the outcome, we examined the cross-correlation between features from CAE and VIs as shown in **Figure 11**. The plot shows relatively high correlations between several VIs and the ze components from the CAE. This overlap may be introducing redundancy, which could be affecting the combined model’s performance. We also note that the CAE currently processes images from different locations based on calendar timepoints. Aligning the images by growth stages, rather than dates, might help improve the consistency of the encoded information and could potentially lead to better model performance in future work.

**Table 7:** Performance on predicting top 25% and top 50% of the genotype for different locations was obtained via a model trained from CAE (compositional autoencoder) latents, VIs (vegetation indices), and CAE + VIs.

Location	VIs	CAE	CAE+VIs
(a) Top 25%			
Ames	44.44	**50.0**	27.77
Crawfordsville	38.33	**50.0**	50.0
Lincoln	50.0	**55.55**	50.0
MOValley	27.78	**55.55**	33.33
Scottsbluff	**44.33**	33.33	38.88
(b) Top 50%			
Ames	56.75	56.75	**67.56**
Crawfordsville	62.16	64.86	**70.27**
Lincoln	37.84	**59.45**	56.75
MOValley	45.94	75.67	**78.37**
Scottsbluff	40.54	**56.75**	51.35

For testing the model for a location, we trained the model on data from all other locations and then predicted yield for all the samples from that location. Then, we rank ordered the predictions and compared them against the ground truth rank ordering of the genotypes. The performance was evaluated by calculating the percentage of overlap between the top 25% or top 50% of the ground truth rank order list and the predicted rank order list. All the numbers given below are in percentages. The numbers in bold represent the best performance observed for a particular location.

#### 3.2.3 Experiment 3: performance on an unseen environment

In the third experiment, we evaluated the CAE’s performance in predicting yield in a completely new environment. To do this, we used data from Ames during the 2023 growing season to test the downstream prediction accuracy of a model that uses the CAE latent representation as inputs. Specifically, a disentangled latent representation was first generated via the CAE on all available (unlabeled) data – three from 2022 and three from 2023 (Ames, Lincoln, and MO Valley). These disentangled latent features were then used to train a downstream XGBoost model to predict yield. The downstream model was trained on all the data except Ames 2023. We then tested this model on Ames 2023 data and observed an RMSE of 27.815, demonstrating an improvement over traditional methods such as linear mixed models (RMSE = 29.91) and vegetation indices (RMSE = 45.80). The CAE also outperformed large pre-trained models like ResNet-18, which had an RMSE of 48. This highlights the ability of the CAE to generalize well to new environmental conditions, even when models like ResNet-18 (He et al., 2016), with its 11.7 million parameters and deeper architecture, struggled.

## 4 Conclusion

This study extends our previous work on disentangling Genotype x Environment (GxE) features by applying a Compositional Autoencoder (CAE) to create disentangled latent representations of satellite imagery. These disentangled latent representations produced improved yield prediction. Our results demonstrate the CAE’s effectiveness in separating environmental factors and improving yield predictions at various growth stages. The CAE outperformed raw satellite data in distinguishing macro-environmental factors, as evidenced by the improvement in silhouette scores from 0.293 to 0.919. This enhanced separation of environmental features suggests the CAE’s potential for more precise modeling of environmental influences on crop performance. The model also showed promising results in disentangling micro-environmental factors.

In terms of yield prediction, regression (XGBoost) models trained on CAE-based latent representations consistently outperformed regression models trained on latent representations from vanilla autoencoders across all time points and beat models trained on VIs for yield prediction for the vegetative stage and post-flowering stage, with competitive performance during the flowering stage. This enhanced early-stage prediction capability could provide breeders with valuable insights for resource management throughout the growing season.

The genotype features extracted by the CAE could also be valuable for genome-wide association studies, offering a new way to link genetic markers to complex traits. Additionally, exploring the CAE’s use with other sensing modalities and applying it to time-series data may further improve its predictive capabilities and reveal new biological insights. Combining the CAE’s disentangled latent representations with other data sources, such as crop models or physiological measurements, could lead to enhanced end-of-season trait prediction models. While this study focused on high-resolution (30 cm) Pleiades Neo imagery to enable fine-grained plot-level yield prediction, an important direction for future work is to evaluate the performance and generalizability of the CAE framework across coarser resolutions (e.g., 1 m, 3 m, and 10 m) to understand the trade-offs between spatial resolution, model accuracy, and operational scalability for broader deployment. Another important direction is to examine how pixel size relates to maize plant size, incorporating factors such as planting density and pixel-level variability, and to assess how these spatial effects correlate with trait variability among maize hybrids.

## Data Availability

All code is available at bitbucket at https://bitbucket.org/baskargroup/cae_sat/src/main/. The satellite plot-level images from multiple time points and locations of experimental fields, along with ground truth data, are accessible at https://doi.org/doi:10.5061/dryad.905qftttm.
