# Supplementary material for: Enhancing yield prediction from plot-level satellite imagery through genotype and environment feature disentanglement
Source: Front Plant Sci. 2025 Sep 30;16:1617831. doi: 10.3389/fpls.2025.1617831 (PMC12518292; doi:10.3389/fpls.2025.1617831)
Supplement: Supplementary file 1 [file DataSheet1.pdf]

Supplementary Material

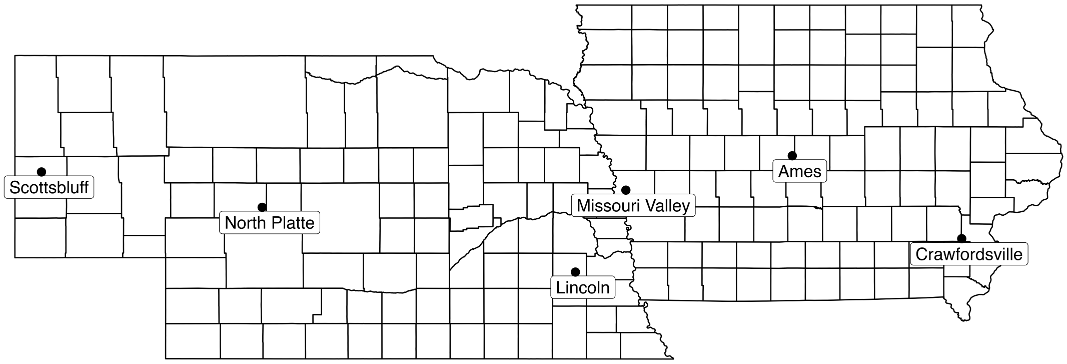

Supplementary Figure 1: Map showing all locations where data used in this study were collected.

1 Vegetation Indices

To avoid division by zero, a small constant $\epsilon = 10^{-6}$ is added:

$$\text{sum\_rgb} = R + G + B + \epsilon$$

The normalized R, G, and B values are calculated as:

$$r = \frac{R}{\text{sum\_rgb}}, \quad g = \frac{G}{\text{sum\_rgb}}, \quad b = \frac{B}{\text{sum\_rgb}}$$

1.1 Green Leaf Index (GLI) [10]

$$\text{GLI} = \frac{2g - r - b}{2g + r + b + \epsilon}$$

GLI measures the relative abundance of green vegetation.

1.2 Normalized Green-Red Difference Index (NGRDI) [13]

$$\text{NGRDI} = \frac{g - r}{g + r + \epsilon}$$

NGRDI assesses the difference between green and red channels, commonly used for vegetation detection.

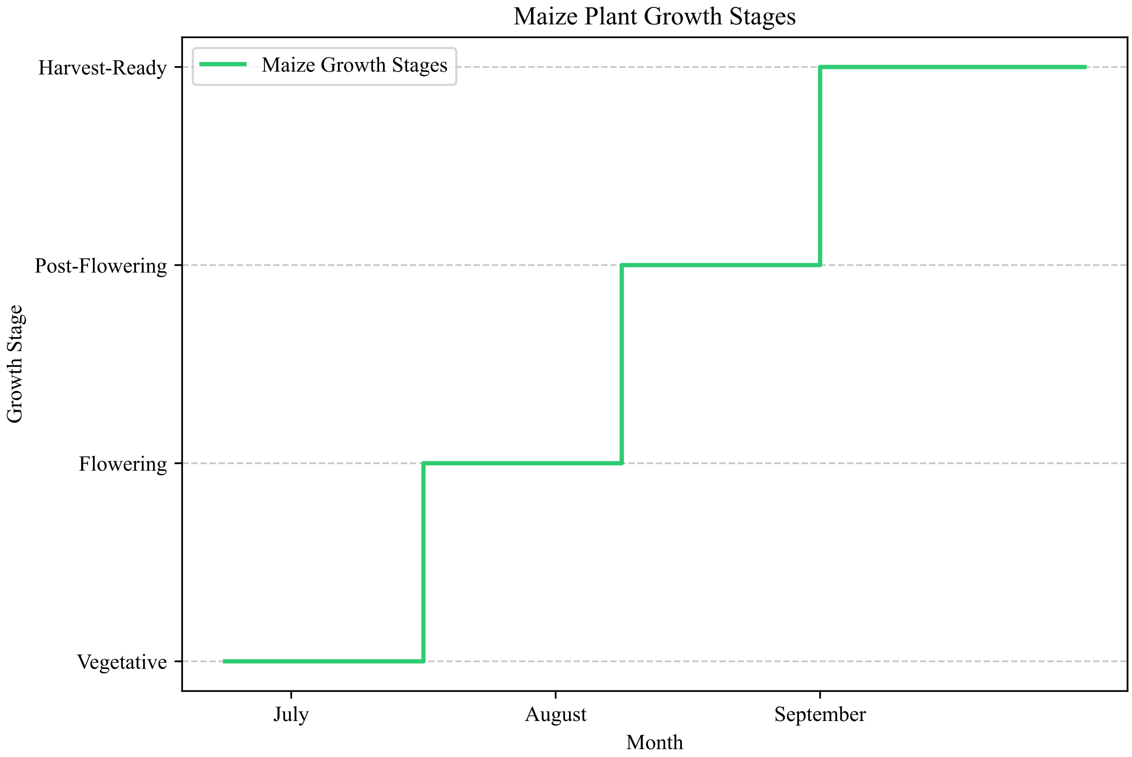

Supplementary Figure 2: Plot showing the growth stages of the maize plants observed for this study for the Lincoln location.

1.3 Visible Atmospherically Resistant Index (VARI) [4]

$$\text{VARI} = \frac{g - r}{g + r - b + \epsilon}$$

VARI is designed to minimize the effects of atmospheric conditions on vegetation detection.

1.4 Vegetative Index (VEG) [5]

$$\text{VEG} = \frac{g}{r^{0.667} \cdot b^{(1-0.667)} + \epsilon}$$

VEG combines the green, red, and blue channels to estimate vegetation.

1.5 Red-Green-Blue Vegetation Index (RGBVI) [1]

$$\text{RGBVI} = \frac{g^2 - (b \cdot r)}{g^2 + (b \cdot r) + \epsilon}$$

RGBVI leverages RGB channels to identify areas with vegetation.

1.6 Excess Green Index (ExG) [8]

$$\text{ExG} = 2g - r - b$$

ExG highlights green vegetation by emphasizing the green channel relative to red and blue.

1.7 Excess Red Index (ExR) [9]

$$\text{ExR} = 1.4r - g$$

ExR enhances the detection of red areas, used for identifying non-vegetated areas.

1.8 Normalized Difference Vegetation Index (NDVI) [12]

$$\text{NDVI} = \frac{NIR - R}{NIR + R + \epsilon}$$

NDVI is one of the most common indices for detecting vegetation using Near Infrared (NIR) and Red (R) bands.

1.9 Green Normalized Difference Vegetation Index (GNDVI) [3]

$$\text{GNDVI} = \frac{NIR - G}{NIR + G + \epsilon}$$

GNDVI is similar to NDVI but uses the green channel instead of the red for vegetation detection.

1.10 Enhanced Vegetation Index (EVI) [6]

$$\text{EVI} = 2.5 \cdot \frac{NIR - R}{NIR + 6R - 7.5B + 1 + \epsilon}$$

EVI improves upon NDVI by correcting for atmospheric conditions and soil background.

1.11 Soil-Adjusted Vegetation Index (SAVI) [7]

$$\text{SAVI} = 1.5 \cdot \frac{NIR - R}{NIR + R + 0.5 + \epsilon}$$

SAVI adjusts NDVI for soil brightness, useful for areas with sparse vegetation.

1.12 Normalized Difference Red Edge (NDRE) [2]

$$\text{NDRE} = \frac{NIR - RE}{NIR + RE + \epsilon}$$

NDRE is similar to NDVI but uses the Red Edge (RE) band, which is sensitive to chlorophyll content.

1.13 Renormalized Difference Vegetation Index (RDVI) [11]

$$\text{RDVI} = \frac{NIR - R}{\sqrt{NIR + R + \epsilon}}$$

RDVI balances NDVI with a normalization factor, providing a different measure of vegetation health.

Supplementary Table 1: Vegetation Indices and Their Formulas

**Index**	**Formula**
GLI	$\frac{2g-r-b}{2g+r+b+\epsilon}$
NGRDI	$\frac{g-r}{g+r+\epsilon}$
VARI	$\frac{g-r}{g+r-b+\epsilon}$
VEG	$\frac{g}{r^{0.667}\cdot b^{(1-0.667)}+\epsilon}$
RGBVI	$\frac{g^2-(b\cdot r)}{g^2+(b\cdot r)+\epsilon}$
ExG	$2g-r-b$
ExR	$1.4r-g$
NDVI	$\frac{NIR-R}{NIR+R+\epsilon}$
GNDVI	$\frac{NIR-G}{NIR+G+\epsilon}$
EVI	$2.5\cdot\frac{NIR-R}{NIR+6R-7.5B+1+\epsilon}$
SAVI	$1.5\cdot\frac{NIR-R}{NIR+R+0.5+\epsilon}$
NDRE	$\frac{NIR-RE}{NIR+RE+\epsilon}$
RDVI	$\frac{NIR-R}{\sqrt{NIR+R}+\epsilon}$

2 Latent configuration sensitivity analysis

Supplementary Table 2: Performance Metrics for CAE with XgBoost across Different Latent Configurations

zg-ze-zp	R^2 (SD)	RMSE (SD) (tons/ha)
3-3-1	0.70 (0.06)	1.60 (0.16)
6-6-1	0.79 (0.03)	1.34 (0.12)
6-6-3	0.79 (0.05)	1.36 (0.14)
6-6-6	0.75 (0.05)	1.49 (0.14)
6-6-12	0.76 (0.04)	1.44 (0.13)
6-6-24	0.73 (0.06)	1.52 (0.17)
12-12-1	0.77 (0.03)	1.41 (0.11)
6-12-1	0.72 (0.04)	1.55 (0.15)
6-24-1	0.74 (0.05)	1.51 (0.16)
24-24-1	0.76 (0.05)	1.44 (0.17)
48-48-1	0.76 (0.05)	1.43 (0.17)

3 Performance of different models across timepoints

Supplementary Table 3: Performance Metrics for TP1 and TP2

Features	Regressor	TP1		TP2	
		R^2 (SD)	RMSE (SD) (tons/ha)	R^2 (SD)	RMSE (SD) (tons/ha)
AE	PLSR	0.46 (0.06)	2.17 (0.12)	0.73 (0.05)	1.54 (0.15)
	XgBoost	0.70 (0.05)	1.61 (0.14)	0.77 (0.06)	1.42 (0.18)
	Ridge	0.45 (0.10)	2.16 (0.18)	0.72 (0.04)	1.54 (0.09)
	Random Forest	0.68 (0.07)	1.64 (0.16)	0.76 (0.05)	1.43 (0.13)
VI	PLSR	0.71 (0.06)	1.59 (0.15)	0.71 (0.75)	1.58 (0.20)
	XgBoost	0.73 (0.05)	1.54 (0.15)	0.79 (0.05)	1.34 (0.16)
	Ridge	0.73 (0.04)	1.51 (0.11)	0.80 (0.04)	1.32 (0.11)
	Random Forest	0.75 (0.04)	1.47 (0.15)	0.80 (0.04)	1.30 (0.09)
CAE	PLSR	0.80 (0.02)	1.30 (0.11)	0.81 (0.04)	1.28 (0.15)
	XgBoost	0.79 (0.03)	1.34 (0.12)	0.80 (0.05)	1.33 (0.16)
	Ridge	0.81 (0.04)	1.26 (0.09)	0.81 (0.04)	1.27 (0.09)
	Random Forest	0.80 (0.05)	1.31 (0.10)	0.81 (0.04)	1.28 (0.09)

Supplementary Table 4: Performance Metrics for TP3 and TP4

Features	Regressor	TP3		TP4	
		R^2 (SD)	RMSE (SD) (tons/ha)	R^2 (SD)	RMSE (SD) (tons/ha)
AE	PLSR	0.10 (0.07)	2.80 (0.17)	0.37 (0.06)	2.36 (0.14)
	XgBoost	0.64 (0.08)	1.77 (0.24)	0.40 (0.09)	2.29 (0.17)
	Ridge	0.08 (0.10)	2.81 (0.18)	0.34 (0.12)	2.37 (0.12)
	Random Forest	0.64 (0.12)	1.73 (0.20)	0.43 (0.09)	2.21 (0.14)
VI	PLSR	-0.05 (0.49)	2.89 (0.59)	0.35 (0.09)	2.38 (0.18)
	XgBoost	0.76 (0.06)	1.43 (0.17)	0.69 (0.07)	1.64 (0.18)
	Ridge	0.21 (0.35)	2.55 (0.53)	-0.19 (1.36)	2.88 (1.26)
	Random Forest	0.77 (0.05)	1.39 (0.15)	0.70 (0.07)	1.57 (0.18)
CAE	PLSR	0.78 (0.04)	1.40 (0.14)	0.49 (0.08)	2.10 (0.17)
	XgBoost	0.77 (0.04)	1.40 (0.14)	0.79 (0.04)	1.37 (0.15)
	Ridge	0.78 (0.04)	1.36 (0.10)	0.52 (0.07)	2.03 (0.15)
	Random Forest	0.79 (0.04)	1.33 (0.12)	0.79 (0.04)	1.35 (0.13)

4 Pairwise ANOVA results and insights

In the context of Analysis of Variance (ANOVA), the **F-statistic** is a measure used to compare the variances between different groups (or treatments) to the variance within those groups. It is calculated as the ratio of the mean square between groups (explaining differences due to the treatments) to the mean square within groups (explaining random variation or error). A higher F-statistic value suggests that the differences between the group means are larger relative to the variation within the groups, indicating potential significant differences.

The **P-value** represents the probability of obtaining the observed data (or more extreme data) assuming the null hypothesis is true (i.e., no real difference between the groups). A common threshold for significance is $p < 0.05$; if the p-value is below this, we reject the null hypothesis and conclude that there is a statistically significant difference between the groups. Conversely, a p-value ≥ 0.05 suggests insufficient evidence to reject the null hypothesis, meaning the difference is not statistically significant.

Supplementary Table 5: Combined Pairwise ANOVA Results for TP1 (R-squared and RMSE)

	R-squared			RMSE		
Pair	F-statistic	P-value	Significant	F-statistic	P-value	Significant
CAE vs CAE+VIs	0.62	0.44	No	0.61	0.44	No
CAE vs VIs	27.34	0.00	Yes	24.86	0.00	Yes
CAE vs VAE	70.45	0.00	Yes	75.24	0.00	Yes
VIs vs VAE	10.36	0.002	Yes	10.35	0.002	Yes

Insights for TP1: The combined results for R-squared and RMSE in TP1 show consistent patterns across metrics. No significant difference between CAE and CAE+VIs in either metric ($p > 0.4$). However, significant differences are observed in CAE vs VIs, CAE vs VAE, and VIs vs VAE, with high F-statistics (e.g., 70.4468 for R-squared in CAE vs VAE), indicating strong distinctions, particularly involving VAE. RMSE results mirror R-squared closely, suggesting robust treatment effects with VAE as the most distinct.

Supplementary Table 6: Combined Pairwise ANOVA Results for TP2 (R-squared and RMSE)

	R-squared			RMSE		
Pair	F-statistic	P-value	Significant	F-statistic	P-value	Significant
CAE vs CAE+VIs	3.04	0.09	No	3.18	0.08	No
CAE vs VIs	0.06	0.81	No	0.05	0.82	No
CAE vs VAE	5.43	0.02	Yes	5.29	0.03	Yes
VIs vs VAE	4.84	0.03	Yes	4.59	0.04	Yes

Insights for TP2: Overall R^2 means show CAE+VIs performing best (0.8204), followed by CAE and VIs, with VAE lowest (0.7495). In pairwise comparisons, no significant differences among CAE, CAE+VIs, and VIs for both metrics ($p > 0.05$), but VAE differs significantly from CAE and VIs ($p < 0.05$), though with lower F-statistics compared to other TPs. This indicates convergence among CAE-related treatments, with VAE as an outlier, consistent across R-squared and RMSE.

Supplementary Table 7: Combined Pairwise ANOVA Results for TP3 (R-squared and RMSE)

	R-squared			RMSE		
Pair	F-statistic	P-value	Significant	F-statistic	P-value	Significant
CAE vs CAE+VIs	0.39	0.54	No	0.39	0.54	No
CAE vs VIs	0.51	0.48	No	0.31	0.58	No
CAE vs VAE	56.35	0.00	Yes	43.61	0.00	Yes
VIs vs VAE	39.72	0.00	Yes	33.79	0.00	Yes

Insights for TP3: Overall R^2 means indicate CAE+VIs highest (0.7802), with CAE and VIs similar, and VAE much lower (0.6326). Pairwise results show no significance among CAE, CAE+VIs, and VIs ($p > 0.2$), but high significance for comparisons with VAE (e.g., F=56.35 for R-squared in CAE vs VAE). RMSE follows suit with slightly lower F-values but same patterns, highlighting VAE's distinct under performance by TP3.

Supplementary Table 8: Combined Pairwise ANOVA Results for TP4 (R-squared and RMSE)

	R-squared			RMSE		
Pair	F-statistic	P-value	Significant	F-statistic	P-value	Significant
CAE vs CAE+VIs	0.72	0.40	No	0.64	0.43	No
CAE vs VIs	34.93	0.00	Yes	31.65	0.00	Yes
CAE vs VAE	352.96	0.00	Yes	365.31	0.00	Yes
VIs vs VAE	166.54	0.00	Yes	164.06	0.00	Yes

Insights for TP4: Overall R^2 means show CAE+VIs best (0.7959), CAE close, VIs lower (0.6894), and VAE poorest (0.3731). Pairwise comparisons reveal no difference between CAE and CAE+VIs ($p > 0.3$), but significant differences in all others, with extremely high F-statistics involving VAE (e.g., 352.9 for R-squared in CAE vs VAE). RMSE metrics align closely, with even higher F for CAE vs VAE (365.3), indicating escalating distinctions by TP4, especially VAE's divergence.

5 Hyperparameter settings for downstream models

The hyperparameters given in the table 9 are the best configurations we found for each of the feature sets. Feature sets here, are the features generated by the three methods (VIs, CAE, and AE) from the satellite data.

Supplementary Table 9: Hyperparameter Settings for Different Feature Representation for the XgBoost Model

features type	max_depth	n_estimators	learning_rate
VIs	5	600	0.01
CAE	2	600	0.01
VAE	3	1000	0.01

6 Data Splits and Evaluation Protocol

For **Experiment 1** (predicting yield for unseen genotypes), we grouped the data according to genotype names and transformed the original NumPy array from shape $N \times F$, where N is the number of samples and F is the number of features, to shape $M \times G \times F$, where G is the number of unique genotypes in the dataset and M is the number of samples per genotype. We then performed 5-fold cross-validation by sampling along the M dimension to create the training and test sets for each fold.

For **Experiment 2** (rank ordering top-yielding genotypes), we extracted all samples from one location and used them as the test set to evaluate performance on data from this *unseen* location (i.e., not seen by the model during training).

For **Experiment 3** (performance in an unseen environment), we followed the same procedure as in Experiment 2, where all samples from the location `Ames_2023` were used as the test set to assess model performance in a new environment.

References

[1] Juliane Bendig et al. "Combining UAV-based plant height from crop surface models, visible, and near infrared vegetation indices for biomass monitoring in barley". In: *International Journal of Applied Earth Observation and Geoinformation* 39 (2015), pp. 79–87. ISSN: 1569-8432. DOI: https://doi.org/10.1016/j.jag.2015.02.012. URL: https://www.sciencedirect.com/science/article/pii/S0303243415000446.

[2] Anatoly Gitelson and Mark N. Merzlyak. "Quantitative estimation of chlorophyll-a using reflectance spectra: Experiments with autumn chestnut and maple leaves". In: *Journal of Photochemistry and Photobiology B: Biology* 22.3 (1994), pp. 247–252. ISSN: 1011-1344. DOI: https://doi.org/10.1016/1011-1344(93)06963-4. URL: https://www.sciencedirect.com/science/article/pii/1011134493069634.

[3] Anatoly A. Gitelson, Yoram J. Kaufman, and Mark N. Merzlyak. "Use of a green channel in remote sensing of global vegetation from EOS-MODIS". In: *Remote Sensing of Environment* 58.3 (1996), pp. 289–298. ISSN: 0034-4257. DOI: https://doi.org/10.1016/S0034-4257(96)00072-7. URL: https://www.sciencedirect.com/science/article/pii/S0034425796000727.

[4] Anatoly A. Gitelson et al. "Novel algorithms for remote estimation of vegetation fraction". In: *Remote Sensing of Environment* 80.1 (2002), pp. 76–87. ISSN: 0034-4257. DOI: https://doi.org/10.1016/S0034-4257(01)00289-9. URL: https://www.sciencedirect.com/science/article/pii/S0034425701002899.

[5] T. Hague, N. D. Tillett, and H. Wheeler. "Automated Crop and Weed Monitoring in Widely Spaced Cereals". In: *Precision Agriculture* 7.1 (Mar. 2006), pp. 21–32. ISSN: 1573-1618. DOI: 10.1007/s11119-005-6787-1. URL: https://doi.org/10.1007/s11119-005-6787-1.

[6] A Huete et al. "Overview of the radiometric and biophysical performance of the MODIS vegetation indices". In: *Remote Sensing of Environment* 83.1 (2002). The Moderate Resolution Imaging Spectroradiometer (MODIS): a new generation of Land Surface Monitoring, pp. 195–213. ISSN: 0034-4257. DOI: https://doi.org/10.1016/S0034-4257(02)00096-2. URL: https://www.sciencedirect.com/science/article/pii/S0034425702000962.

[7] A.R Huete. "A soil-adjusted vegetation index (SAVI)". In: *Remote Sensing of Environment* 25.3 (1988), pp. 295–309. ISSN: 0034-4257. DOI: https://doi.org/10.1016/0034-4257(88)90106-X. URL: https://www.sciencedirect.com/science/article/pii/003442578890106X.

[8] D. M. Woebbecke et al. "Color Indices for Weed Identification Under Various Soil, Residue, and Lighting Conditions". In: *Transactions of the ASAE* 38.1 (1995), pp. 259–269. ISSN: 0001-2351. URL: https://elibrary.asabe.org/abstract.asp?aid=27838&t=3.

[9] George E. Meyer and João Camargo Neto. "Verification of color vegetation indices for automated crop imaging applications". In: *Computers and Electronics in Agriculture* 63.2 (2008), pp. 282–293. ISSN: 0168-1699. DOI: https://doi.org/10.1016/j.compag.2008.03.009. URL: https://www.sciencedirect.com/science/article/pii/S0168169908001063.

[10] Michael M. Borman Mounir Louhaichi and Douglas E. Johnson. "Spatially Located Platform and Aerial Photography for Documentation of Grazing Impacts on Wheat". In: *Geocarto International* 16.1 (2001), pp. 65–70. DOI: 10.1080/10106040108542184. eprint: https://doi.org/10.1080/10106040108542184. URL: https://doi.org/10.1080/10106040108542184.

[11] Jean-Louis Roujean and François-Marie Breon. "Estimating PAR absorbed by vegetation from bidirectional reflectance measurements". In: *Remote Sensing of Environment* 51.3 (1995), pp. 375–384. ISSN: 0034-4257. DOI: https://doi.org/10.1016/0034-4257(94)00114-3. URL: https://www.sciencedirect.com/science/article/pii/0034425794001143.

[12] John Wilson Rouse et al. "Monitoring vegetation systems in the Great Plains with ERTS". In: *NASA Spec. Publ* 351.1 (1974), p. 309.

[13] Compton J. Tucker. "Red and photographic infrared linear combinations for monitoring vegetation". In: *Remote Sensing of Environment* 8.2 (1979), pp. 127–150. ISSN: 0034-4257. DOI: https://doi.org/10.1016/0034-4257(79)90013-0. URL: https://www.sciencedirect.com/science/article/pii/0034425779900130.
